# Utilizing metagenomic next-generation sequencing for diagnosis and lung microbiome probing of pediatric pneumonia through bronchoalveolar lavage fluid in pediatric intensive care unit: results from a large real-world cohort

**DOI:** 10.3389/fcimb.2023.1200806

**Published:** 2023-08-15

**Authors:** Huili Shen, Tingyan Liu, Meili Shen, Yi Zhang, Weiming Chen, Hanlin Chen, Yixue Wang, Jing Liu, Jinhao Tao, Liming He, Guoping Lu, Gangfeng Yan

**Affiliations:** ^1^ Pediatric Intensive Care Unit, Children’s Hospital of Fudan University, National Children’s Medical Center, Shanghai, China; ^2^ Medical Department, Nanjing Dinfectome Technology Inc., Nanjing, Jiangsu, China; ^3^ Department of Clinical Epidemiology, Children’s Hospital of Fudan University, National Children’s Medical Center, Shanghai, China

**Keywords:** bronchoalveolar lavage fluid, metagenomic next-generation sequencing, pneumonia, diagnosis, lung microbiome, pediatric intensive care unit

## Abstract

**Background:**

Metagenomic next-generation sequencing (mNGS) is a powerful method for pathogen detection in various infections. In this study, we assessed the value of mNGS in the pathogen diagnosis and microbiome analysis of pneumonia in pediatric intensive care units (PICU) using bronchoalveolar lavage fluid (BALF) samples.

**Methods:**

A total of 104 pediatric patients with pneumonia who were admitted into PICU between June 2018 and February 2020 were retrospectively enrolled. Among them, 101 subjects who had intact clinical information were subject to parallel comparison of mNGS and conventional microbiological tests (CMTs) for pathogen detection. The performance was also evaluated and compared between BALF-mNGS and BALF-culture methods. Moreover, the diversity and structure of all 104 patients’ lung BALF microbiomes were explored using the mNGS data.

**Results:**

Combining the findings of mNGS and CMTs, 94.06% (95/101) pneumonia cases showed evidence of causative pathogenic infections, including 79.21% (80/101) mixed and 14.85% (15/101) single infections. Regarding the pathogenesis of pneumonia in the PICU, the fungal detection rates were significantly higher in patients with immunodeficiency (55.56% vs. 25.30%, P =0.025) and comorbidities (40.30% vs. 11.76%, P=0.007). There were no significant differences in the α-diversity either between patients with CAP and HAP or between patients with and without immunodeficiency. Regarding the diagnostic performance, the detection rate of DNA-based BALF-mNGS was slightly higher than that of the BALF-culture although statistically insignificant (81.82% vs.77.92%, P=0.677) and was comparable to CMTs (81.82% vs. 89.61%, P=0.211). The overall sensitivity of DNA-based mNGS was 85.14% (95% confidence interval [CI]: 74.96%-92.34%). The detection rate of RNA-based BALF-mNGS was the same with CMTs (80.00% vs 80.00%, P>0.999) and higher than BALF-culture (80.00% vs 52.00%, P=0.045), with a sensitivity of 90.91% (95%CI: 70.84%-98.88%).

**Conclusions:**

mNGS is valuable in the etiological diagnosis of pneumonia, especially in fungal infections, and can reveal pulmonary microecological characteristics. For pneumonia patients in PICU, the mNGS should be implemented early and complementary to CMTs.

## 1 Introduction

Pneumonia is a leading cause of morbidity and mortality in children worldwide, especially those under 5 years old (Rudan et al., 2008; Jain et al., 2015). According to a report by the World Health Organization (WHO), pneumonia killed 740 180 children, accounting for 14% of all deaths under 5 years old in 2019 (https://www.who.int/en/news-room/fact-sheets/detail/pneumonia). In mainland China, pneumonia incidence under 5 years old ranged from 0.06–0.27 episodes per person-year, and mortality ranged from 184–1223 deaths per 100,000 population (Guan et al., 2010). Pneumonia can be classified as either community-acquired pneumonia (CAP), and hospital-acquired pneumonia (HAP), which may be ventilator-associated pneumonia (VAP) or acquired in the absence of mechanical ventilation (Sandora and Harper, 2005). About 12-20% of pediatric CAP cases require critical care (Dassner et al., 2017). The estimated incidence of VAP in pediatric intensive care units (PICU) ranges from 1.8 to 8.3 per 1000 ventilator days (Kohbodi et al., 2022). Because of the heavy burden of pneumonia on children’s health, mortality, and costs (Walker et al., 2013), the optimal management of pneumonia, especially the determination of microbial etiology, is a hot topic of considerable importance. The microbial etiology in pediatric pneumonia is varied by acquired source, immune status, comorbidity, and age. For instance, the causes of pneumonia in the immunodeficient hosts with different types and severities of immunodeficiency consist of different opportunistic agents, apart from regular agents (Eslamy and Newman, 2011). These immunodeficient host should be considered at high risk for infection and merits a more aggressive diagnostic and therapeutic approach (Sandora and Harper, 2005).

Significant technological advances, particularly in molecular diagnostics, have increased the potential to identify and characterize the roles of both existing and previously unrecognizing pneumonia pathogens, but the clinical yield of pathogens is not yet satisfactory. According to a systematic review, the yield of pathogens among children was 52.0% ± 18.1%, similar to adults of 51.0% ± 17.9% (Zhu et al., 2018). As a representative of modern molecular technology, mNGS is a method for parallel high-throughput sequencing of bacteria, viruses, fungi, parasites, atypical pathogens, and even new microorganisms in one sample (Miller and Chiu, 2021), with a turnaround time of only 24-48h or less (Gu et al., 2021).

To our best knowledge, only a few studies reported the application of BALF-mNGS in pathogen detection in children with pneumonia, which were mainly focused on CAP (Zinter et al., 2019; Wang et al., 2020; Deng et al., 2022; Guo et al., 2022; Yang et al., 2022). However, HAP was more prevalent in the PICU. Meanwhile, BALF-mNGS is a proven technology in revealing the microbiome in pediatric pneumonia (Chiu and Miller, 2019; Chen et al., 2021; Zhou et al., 2022), which can serve as an alternative angle describing the causes and characteristics of pneumonia. Here, we performed a single-center retrospective study to evaluate the clinical performance of mNGS pathogen detection capabilities when using BALF specimens from pneumonia patients in a pediatric intensive care unit, and microbiomes in pediatric pneumonia were evaluated.

## 2 Materials and methods

### 2.1 Patient enrolment

A total of 104 pediatric patients with pneumonia from the pediatric intensive care unit (PICU) of the Children’s Hospital of Fudan University between June 2018 and February 2020 were retrospectively enrolled in this study. The study was performed in accordance with the declaration of Helsinki and was approved by the ethics committee of the Children’s Hospital of Fudan University (Approval ID: 2022-398).

The enrollment criteria for pneumonia were as follows: (i) Manifestations of fever, cough, expectoration, refusal to eat, lethargy, irritability, wheezing, dyspnea, and others; (ii) The respiratory rate increased: the respiratory rate ≥ 60 times/min in patients less than 2 months of age; respiratory rate ≥ 50 times/min between 2 months and 1 year old; respiratory rate ≥ 40 times/min in patients of 1-5 years old; respiratory rate ≥ 30 times/min in patients over 5 years old; (iii) On physical examination of pulmonary signs, mild dullness may be observed on percussion, and fine wet rales or crepitations may be heard; (iv) Imaging of the lungs showed patchy exudation.

The exclusion criteria were as follows: (i)≤ 28 days or > 18 years of age; (ii) Non-infectious factors, such as congenital heart disease, pulmonary edema, asthma, upper airway obstruction, or pulmonary cystic fibrosis; (iii) Contraindications to fiberoptic bronchoscopy, such as patients with severe cardiopulmonary dysfunction and coagulation dysfunction; (iv) Incomplete clinical data; (v) Undetermined prognosis and clinical outcomes.

The final clinical composite diagnosis was decided by two experienced clinical experts jointly according to clinical characteristics and the results of mNGS and CMTs. In cases of inconsistent diagnosis, a third expert would adjudicate.

### 2.2 Sample collection

Bronchoalveolar lavage fluid (BALF) of all patients was collected according to the recommendations of the European Respiratory Society (de Blic et al., 2000). Sedation and topical anesthesia were administered, and age-appropriate pediatric flexible fiberoptic bronchoscopes were selected. More severely diseased regions in patients with diffuse lung disease or the right middle lobes were selected, based on radiological findings or evidence from bronchoscopy. Warm saline (1 mL/kg body weight, maximum 20 mL per fraction) was dripped into the selected lung lobes, and at least 40% of the fluid was recovered by mechanical suction using a pressure of approximately 50 to 100 mmHg. Permission was obtained from the patient’s parents, and written informed consent was obtained prior to BALF collection. Sputum, throat swabs, peripheral blood, urine, anal swabs, and other sample types were also collected for conventional microbiological tests.

### 2.3 Clinical characteristics definition and classification

The definition of Severe pneumonia was referred to the world health organization (WHO) diagnostic criteria: (i)The primary diagnostic criteria were invasive mechanical ventilation, fluid-refractory shock, an urgent need for noninvasive positive-pressure ventilation, and hypoxemia requiring an FiO2 greater than the inhalation concentration or flow rate feasible within general care. (ii)The secondary criteria were an increased respiratory rate, PaO2/FiO2 ratio of < 250, multi-lobar infiltration, Pediatric Early Warning Score > 6, altered mental status, hypotension, presence of effusion, comorbidities (e.g. immunosuppression, immunodeficiency), and unexplained metabolic acidosis.

All the patients were conducted with metagenomic next-generation sequencing (mNGS) and conventional microbiological tests (CMTs). The patients were divided into community-acquired pneumonia (CAP, pneumonia developed outside the hospital and pneumonia developed after admission due to an outside pathogenic infection with a defined incubation period) and hospital-acquired pneumonia (HAP, pneumonia developed 48 hours after admission). The definition of immunodeficiency was a failure or absence of elements of the immune system, including lymphocytes, phagocytes, and the complement system. In this study all patients with immunodeficiency was primary immunodeficiency, which was subdivided into types that cause T-cell deficiency, B-cell deficiency, both T-cell and B-cell deficiency, complement deficiency, phagocyte deficiency, and immunoglobulin A deficiency (Justiz Vaillant and Qurie, 2022).

### 2.4 Conventional microbiological tests(CMTs)

All the samples were subjected to a series of CMTs, which is the current clinical gold standard in pathogen detection. Concretely, CMTs included smears, cultures (including BALF, sputum, blood, urine, throat swabs and other infected lesion specimens), PCR of blood and throat swabs (for *herpes simplex virus, human gammaherpesvirus 4*, and *human betaherpesvirus 5, respiratory syncytial virus, influenza A/B virus, parainfluenza virus, adenovirus, rotavirus, rhinovirus, Mycoplasma pneumoniae*, and *Chlamydia pneumoniae*), and serology tests of blood (including G test, GM test, and antibody detection of *parvovirus, herpes simplex virus, human gammaherpesvirus 4, and human betaherpesvirus 5, respiratory syncytial virus, influenza A/B virus, parainfluenza virus, adenovirus, Mycoplasma pneumoniae, Chlamydia pneumoniae*, and *Legionella pneumophila*).

### 2.5 Metagenomic next-generation sequencing (mNGS) and bioinformatics analysis

DNA was extracted from 300 μL samples using the TIANamp micro DNA kit (TIANGEN Biotech, Beijing, China). RNA was extracted from 300 μL samples using the TIAamp virus RNA Mini kit (QIAGEN, Germany). Then, RNA was reverse-transcribed to cDNA with RNA Super Script II reverse transcriptase (Thermo Fischer Technology, Waltham, Massachusetts, USA). Finally, double-stranded DNA was further synthesized using DNA polymerase I (Enzymatics, USA).

DNA libraries were constructed by fragmentation, end-repair, adaptor ligation, and PCR amplification under the following conditions: 98°C for 2 minutes, followed by 12 cycles of 98°C for 15 s, 56°C for 15 s, and 72°C for 30 s, with a final extension at 72°Cfor 5 minutes. The Agilent 2100 Bioanalyzer system (Agilent, Santa Clara, CA, USA) and the Qubit dsDNA HS Assay Kit (Thermo Fisher Scientific Inc., Waltham, MA, USA) was used to control the DNA library fragment sizes and concentrations. Sequencing was performed according to the manufacturer’s manual as follows: first, the library was thermally denatured to form single-stranded DNA, which was circularized to form a single-stranded circular structure; second, the DNA was amplified using rolling circle amplification technology to form a DNA Nano Ball (DNB, DNA Nano Ball); finally, we completed sequencing in the single-end 50 bp sequencing mode using BGISEQ-50 (MGI Technology, Shenzhen, China).

The bioinformatics analysis was completed by referring to our previously published studies (Yan et al., 2021). Briefly, we removed the low-quality reads with sequence lengths less than 35 bp using the Trimmomatic software (version 0.39). Then BWA software (version 0.7.15-r1140) was used to align the high-quality reads to the human reference genome (version hg19) to remove contamination by human sequences. Duplicate and low complexity reads were removed with Prinseq software (version 0.20.4). Then, the remaining clean reads were blasted to the pathogen database, including 4945 viruses and 6039 bacteria (excluding Mycobacteria) related to human diseases, 174 species of mycobacterium, 137 species of mycoplasma, 1064 species of fungus, and 234 species of protozoa. The principle of reporting positive is: (i) The number of strictly aligned reads for bacteria (except for *Mycobacterium tuberculosis*), fungi, and DNA viruses is greater than 3; (ii) The number of strictly aligned reads for parasites is greater than 100; (iii) Strictly aligned reads for *Mycobacterium tuberculosis* and RNA viruses is greater than 1.

### 2.6 Statistical analysis

The Shannon index and the Simpson index were used to measure the alpha diversity. Student’s t-test or Kruskal–Wallis test was used to analyze the normally distributed or non-normal distribution data. Categorical variables were expressed as percentages (%), and statistical tests were performed using the Chi-squared test or Fisher’s exact test. The “cor.test” function in “stats” R package was used to assess spearman’s correlations between clinical characteristics and the relative abundances of the species. The SPSS 23.0 software (IBM, Armonk, NY, USA.) was used for other data analysis. All P-values were FDR adjusted, and a two-tailed P value < 0.05 was considered statistically significant.

## 3 Results

### 3.1 Patient enrolment and clinical characteristics

**Figure 1:**
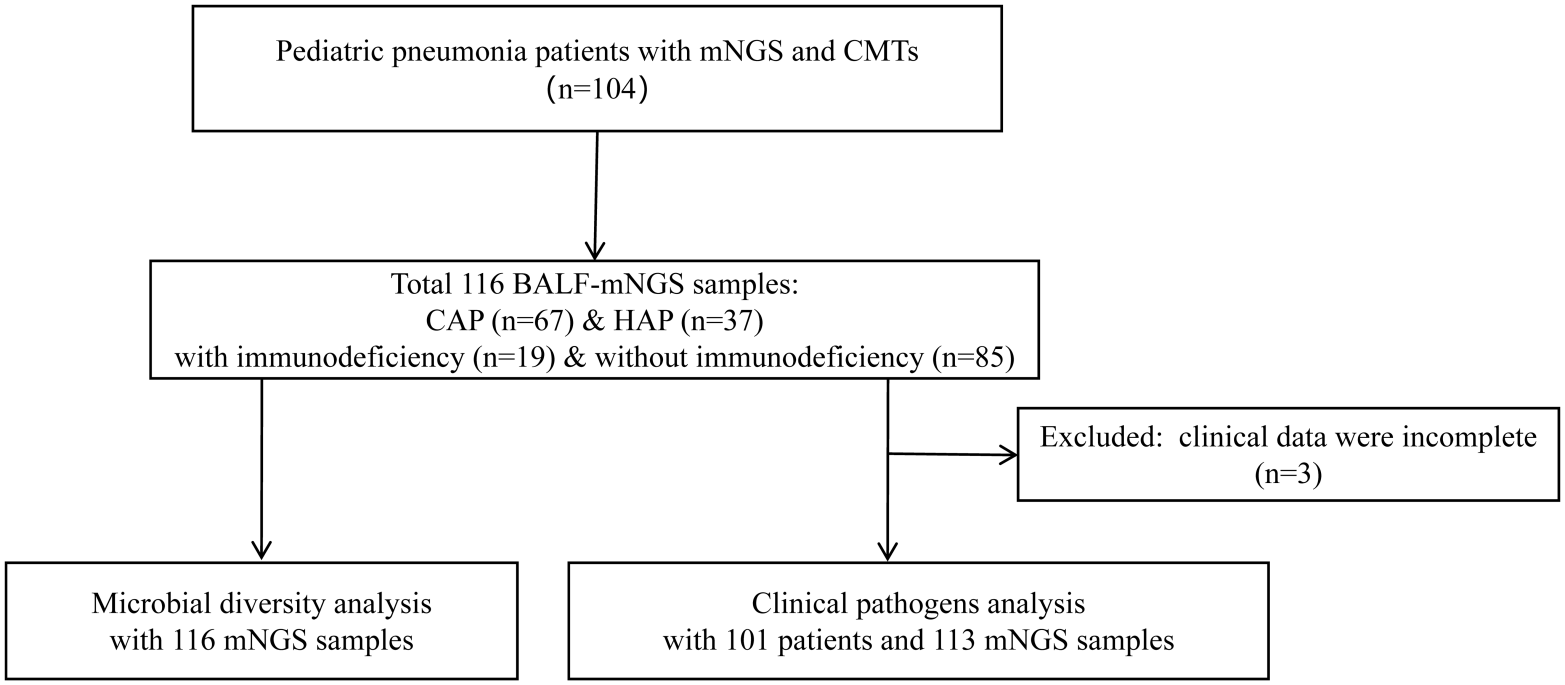
Flow diagram of this study. BALF, Bronchoalveolar lavage fluid; mNGS, Metagenomic next-generation sequencing; CMTs, Conventional microbiological tests; CAP, Community-acquired pneumonia; HAP, Hospital-acquired pneumonia.

A total of 104 children with pneumonia were retrospectively enrolled. As shown in **Figure 1**, 116 BALF samples from 104 patients were collected for mNGS, including 25 for RNA-based mNGS and 91 for DNA-based mNGS based on their clinical manifestations. The BALF samples of 12 patients were collected twice for disease surveillance. Three children were excluded for incomplete clinical data, and finally 101 children underwent clinical pathogens diagnosis combining mNGS and CMTs for pathogenetic. In 101 patients with complete clinical information, all of them underwent culture and CMT testing, while 77 cases used DNA-based mNGS and 25 cases used RNA-based mNGS. One case used DNA and RNA-based mNGS in combination.

The baseline characteristics of enrolled 101 children for clinical pathogens diagnosis are shown in **Table 1**. Of all 101 children, 60.40% (61/101) were males, and 50.50% (51/101) with ages <1 year. There were 67 community-acquired pneumonia cases and 37 with hospital-acquired pneumonia. 17.82% (18/101) of children were immunodeficient, 66.34% (67/101) of children had comorbidities, and 29.70% (30/101) were diagnosed with severe pneumonia. 89 out of 101 (88.12%) children received mechanical ventilation, and 70 out of 101 (69.31%) children stayed in PICU longer than 14 days. 85.15% (86/101) of children received anti-infective treatments before sampling, and 40.59% (40/101) cases adjusted anti-infective treatments after mNGS. In total, 68.32% (69/101) of children survived and 31.68% (32/101) died.

**Table 1:** Baseline characteristics of 101 pediatric patients for clinical pathogens analysis.

Characteristics	N	%
Age(year)		
<1	51	50.50%
≥1	50	49.50%
Gender		
Male	61	60.40%
Female	40	39.60%
Source of pneumonia		
Community-acquired pneumonia	64	63.37%
Hospital-acquired pneumonia	37	36.63%
Severe pneumonia		
Yes	30	29.70%
No	71	70.30%
Comorbidities		
Yes	67	66.34%
No	34	33.66%
Immunodeficiency		
Yes	18	17.82%
No	83	82.18%
PICU stay		
<14	31	30.69%
≥14	70	69.31%
Mechanical ventilation		
Yes	89	88.12%
No	12	11.88%
Anti-infective treatments before sampling		
Yes	86	85.15%
No	15	14.85%
Adjustments of anti-infective treatment after mNGS		
Yes	41	40.59%
No	60	59.41%
Outcomes		
Survived	69	68.32%
Died	32	31.68%

### 3.2 Diagnostic performance of mNGS and CMTs in pediatric pneumonia patients

**Figure 2:**
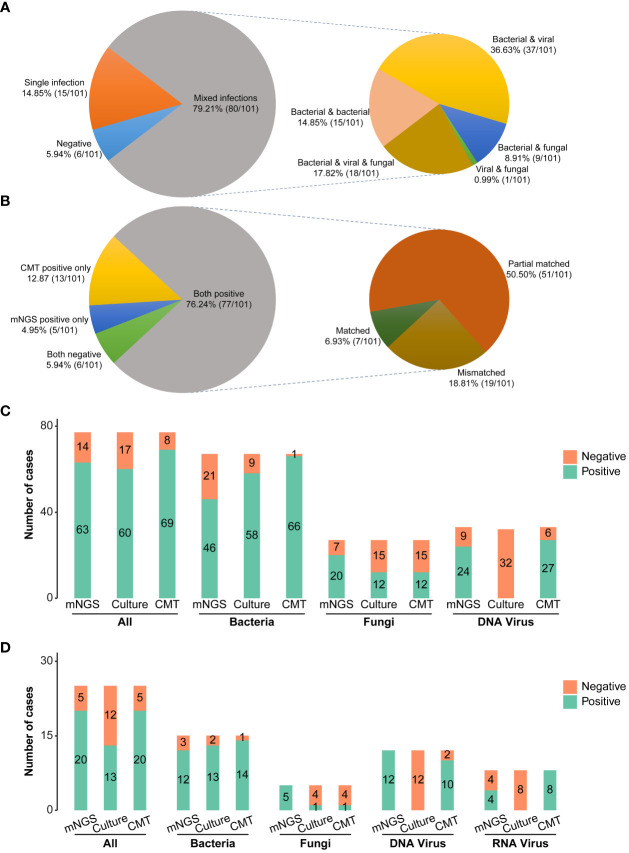
Pathogen detection comparations among mNGS, conventional microbiological tests, and culture. **(A)** Distribution of single and mixed infection types in 101 patients. **(B)** Concordance of diagnosis results between mNGS and CMTs **(C)** Bar plots showing the numbers of bacteria, fungi, and virus infections detected by DNA mNGS, CMTs, and culture. **(D)** Bar plots showing the numbers of bacteria, fungi, and virus infections detected by RNA mNGS, CMTs, and culture. mNGS, Metagenomic next-generation sequencing; CMTs, Conventional microbiological tests; CAP, Community-acquired pneumonia; HAP, Hospital-acquired pneumonia.

The clinical composite diagnosis was used as the reference standard in diagnostic performance evaluations for all methods. As shown in **Figure 2B**, the mNGS and CMTs were both positive in 77 (77/101, 76.24%) patients. A total of 5 (5/101, 4.95%) patients were positive by mNGS only, and 13 (13/101, 12.87%) patients were positive by CMTs only. Additionally, the results between mNGS and CMTs were matched in 7 (7/101, 6.93%) cases, partially matched in 51 (51/101, 50.50%) cases, and mismatched in 19 (19/101, 18.81%) cases.

**Figure 3:**
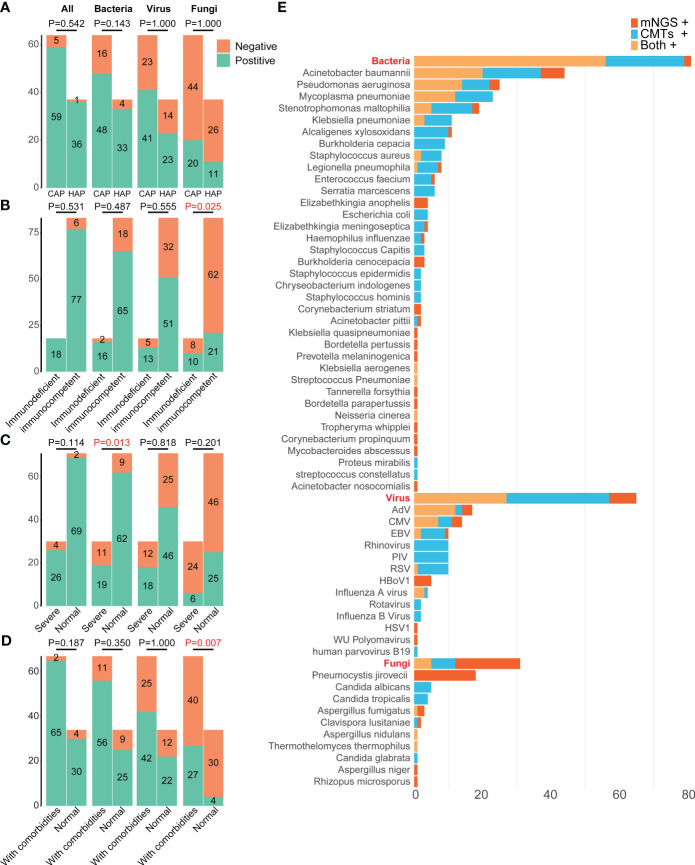
Distributions of pathogens in 101 pediatric pneumonia patients from different subgroups. The differences in the positive rates of bacteria, fungi, virus, and all pathogens between patients **(A)** carrying CAP and HAP, **(B)** with and without immunodeficient, **(C)** with and without severe pneumonia, **(D)** with and without comorbidities. **(E)** Distributions of pathogens detected by mNGS and CMTs. mNGS, Metagenomic next-generation sequencing; CMTs, Conventional microbiological tests; CMV, Human betaherpesvirus 5; EBV, Epstein-barr virus; RSV, Respiratory syncytial virus; HSV1, Human alphaherpesvirus 1; PIV, Parainfluenza virus; HBoV1, Human bocavirus 1; CAP, Community-acquired pneumonia; HAP, Hospital-acquired pneumonia.

**Figure 4:**
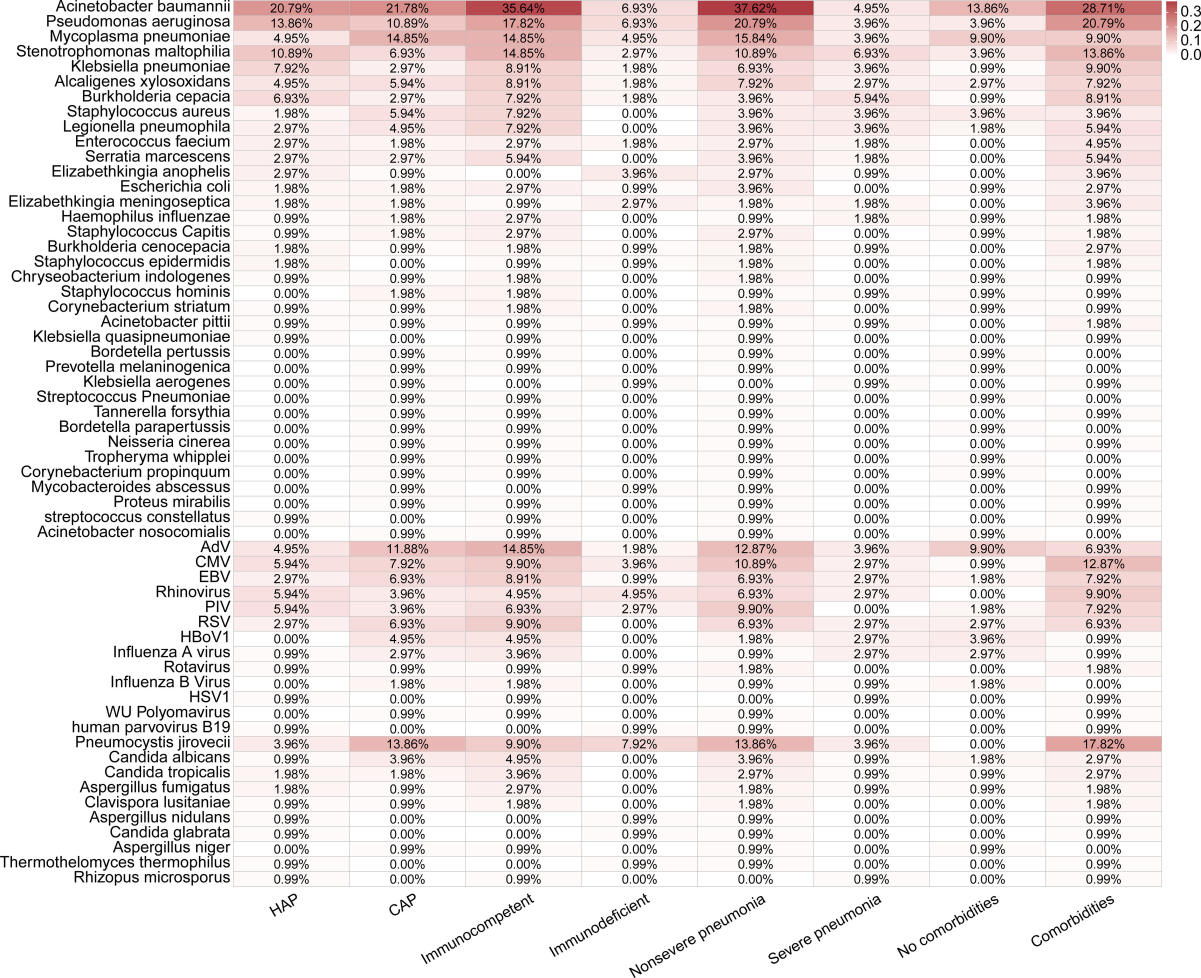
Distributions of pathogens detected in 101 pediatric pneumonia patients in different subgroups.

A total of 59 pathogens were detected in 101 cases by mNGS and CMTs, including 36 bacteria, 13 viruses, and 10 fungi (**Figures 3E**, **4**). More bacterial pathogens were detected by CMTs, especially for common respiratory opportunistic bacteria (**Figure 3E**). Rare bacterial pathogens, including *Bordetella pertussis* and *Bordetella parapertussis*, were only detected by mNGS. Regarding viral pathogens, *AdV, CMV, EBV, Rhinovirus, PIV*, and *RSV* were the most common viruses. DNA viruses, such as *HBoV1*, were only detected by mNGS, while most RNA viruses were detected by CMTs (PCR and/or serological tests).

*Pneumocystis jirovecii* was the most dominant fungal pathogen (58.06%, 18/31), followed by *Candida* sp. (32.26%,10/31) and *Aspergillus* sp. (16.13%, 5/31). All 18 *Pneumocystis jirovecii* cases were detected only by mNGS, while all *Candida* sp. were positive only in CMTs.

The diagnostic performances of mNGS, culture, and CMTs were presented in **Table 2**. For 77 patients who underwent the DNA-based BALF mNGS, its sensitivity was 85.14% (95% CI: 74.96% to 93.24%). The sensitivity of CMTs and culture were 93.24% (95% CI: 84.93% to 97.77%) and 81.08% (95% CI: 70.30% to 89.25%), respectively. The specificity and positive predictive value (PPV) of DNA-based mNGS, culture, and CMTs were all 100.00%. The negative predictive value (NPV) of DNA-based mNGS, culture, and CMTs were 21.43% (95% CI: 4.66% to 50.80%), 17.65% (95% CI: 3.80% to 43.43%), and 37.50% (95% CI: 8.52% to 75.51%), respectively. While for 25 samples underwent RNA-based mNGS, the sensitivity of RNA-based mNGS, culture and CMTs were 90.91% (95% CI: 70.84% to 98.88%), 59.10% (95% CI: 36.35% to 79.29%) and 90.91% (95% CI: 70.84% to 98.88%), respectively. The specificity and positive predictive value (PPV) of RNA-based mNGS, culture, and CMTs were all 100.00% as well. The negative predictive value (NPV) of RNA-based mNGS, culture, and CMTs were 60.00% (95% CI: 14.66% to 94.73%), 25.00% (95% CI: 5.49% to 57.19%), and 60.00% (95% CI: 14.66% to 94.73%), respectively.

**Table 2:** Diagnostic performance of mNGS, culture and CMTs in pediatric pneumonia.

77 cases with both DNA-based mNGS and CMTs							
		Positive(n=74)	Negative (n=3)	sensitivity, % (95%CI)	specificity, % (95%CI)	PPV, % (95%CI)	NPV, % (95%CI)
CMTs	Positive	69	0	93.24% (84.93%, 97.77%)	100.00% (29.24%,100.00%)	100.00% (94.79%, 100.00%)	37.50% (8.52%, 75.51%)
	Negative	5	3				
Culture	Positive	60	0	81.08% (70.30%, 89.25%)	100.00% (29.24%,100.00%)	100.00% (91.04%, 100.00%)	17.65% (3.80%,43.43%)
	Negative	14	3				
mNGS	Positive	63	0	85.14% (74.96%, 92.34%)	100.00% (29.24%, 100.00%)	100.00% (94.13%,100.00%)	21.43% (4.66%,50.80%)
	Negative	11	3				
25 cases with both RNA-based mNGS and CMTs							
		Positive(n=22)	Negative (n=3)	sensitivity, % (95%CI)	specificity, % (95%CI)	PPV, % (95%CI)	NPV, % (95%CI)
CMTs	Positive	20	0	90.91% (70.84%, 98.88%)	100.00% (29.24%, 100.00%)	100.00% (83.16%, 100.00%)	60.00% (14.66%, 94.73%)
	Negative	2	3				
Culture	Positive	13	0	59.10% (36.35%, 79.29%)	100.00% (29.24%, 100.00%)	100.00% (75.29%, 100.00%)	25.00% (5.49%, 57.19%)
	Negative	9	3				
mNGS	Positive	20	0	90.91% (70.84%, 98.88%)	100.00% (29.24%, 100.00%)	100.00% (83.16%, 100.00%)	60.00% (14.66%, 94.73%)
	Negative	2	3				

In 95 pediatric pneumonia patients with specific positive pathogen results, the diagnostic performances of mNGS, BALF-culture, and CMTs for mixed infections were presented in **Table 3**. The sensitivity and specificity of diagnosing mixed infections by mNGS were 53.75% (95% CI: 42.24% - 64.97%) and 100.00% (95% CI: 78.20% - 100.00%), respectively, with PPV and NPV being 100.00% (95% CI: 91.78% - 100.00%) and 28.85% (95% CI: 17.13% - 43.08%). The sensitivity and specificity of diagnosing mixed infection by CMTs were 88.75% (95% CI: 79.72% - 94.72%) and 100.00% (95% CI: 78.20% - 100.00%), respectively, with PPV and NPV being 100.00% (95% CI: 94.94% - 100.00%) and 62.50% (95% CI: 40.59% - 81.20%). The sensitivity and NPV of diagnosing mixed infection by BALF-culture were 60.00% (95% CI: 48.44% - 70.80%) and 31.91% (95% CI: 19.09% - 47.12%), respectively, with the specificity and PPV being both 100.00%.

**Table 3:** Diagnostic performance of mNGS, culture and CMTs for mixed infection in pediatric pneumonia.

		Patients with Positive Infection Pathogens(n=95)					
		Mixed infection (n=80)	Simple infection (n=15)	Sensitivity, % (95%CI)	Specificity, % (95%CI)	PPV, % (95%CI)	NPV, % (95%CI)
mNGS	Positive	43	0	53.75% (42.24%, 64.97%)	100.00% (78.20%,100.00%)	100.00% (91.78%, 100.00%)	28.85% (17.13%, 43.08%)
	Negative	37	15				
Culture	Positive	48	0	60.00% (48.44%, 70.80%)	100.00% (78.20%, 100.00%)	100.00% (92.60%, 100.00%)	31.91% (19.09%, 47.12%)
	Negative	32	15				
CMTs	Positive	71	0	88.75% (79.72%, 94.72%)	100.00% (78.20%, 100.00%)	100.00% (94.94%, 100.00%)	62.50% (40.59%,81.20%)
	Negative	9	15				

### 3.3 Clinical infections and pathogens distribution in pediatric pneumonia patients

Combining the findings of mNGS and CMTs, 94.06% (95/101) pediatric pneumonia patients showed evidence of causative pathogenic infections, including 79.21% (80/101) mixed infections and 14.85% (15/101) single infections (**Figure 2A**). 15 single infections consisted of 4 bacterial infections, 8 viral infections, and 3 fungal infections. Bacteria and viruses (37/101, 36.63%) were the most common mixed infection combination, followed by bacteria and viruses and fungi (18/101, 17.82%), bacteria and bacteria (15/101,14.85%), bacteria and fungi (9/101, 8.91%), viruses and fungi (1/101, 0.99%) (**Figure 2A**). The percentages of patients with bacterial, viral, and fungal infections were 80.20% (81/101), 63.37% (64/101), and 30.69% (31/101), respectively. Of 81 bacterial infections, 30 (30/101, 29.70%) cases were caused by atypical pathogens, including 23 *Mycoplasma pneumoniae*, 1 *Mycobacteroides abscessus*, and 8 *Legionella pneumophila*.

There were no significant differences in the total positive rates between the CAP (92.19%, 59/64) and HAP groups (97.30%, 36/37) (P =0.542, **Figure 3A**). The bacterial positive rates in the HAP groups were higher than that in the CAP groups, although there was no significant difference (89.19% vs. 75.00%, P =0.143), and there were no differences in viral and fungal positive rates between the HAP groups and the CAP groups (viral: 62.16% vs. 64.06%, P =1.000, fungal: 29.73% vs. 31.25%, P =1.000). The total positive rates were 100.00% (18/18) in immunodeficient patients and 92.77% (77/83) (P =0.531, **Figure 3B**). The bacterial, viral, and fungal positive rates of patients with immunodeficiency were all higher than those without immunodeficiency, without statistical significance (**Figure 3B**). The fungal detection rates were significantly higher in immunodeficient patients (55.56% vs. 25.30%, P =0.025, **Figure 3B**), while the bacterial detection rates were higher in non-severe pneumonia patients (87.32% vs. 63.33%, P=0.013, **Figure 3C**). Moreover, the fungal detection rates were significantly higher in the patients with comorbidities (40.30% vs. 11.76%, P =0.007, **Figure 3D**).

Considering different methods for pathogenic detection, the positive rates of mNGS, culture, and CMTs were analyzed in 77 DNA-based and RNA-based mNGS cases, respectively. In 77 DNA-based mNGS cases, the overall positive rates of DNA-based mNGS, culture, and CMTs were 81.82% (63/77), 77.92% (60/77), and 89.61% (69/77), respectively (**Figure 2C**), and there was a significant difference in between CMTs and culture (CMTs vs. culture: 89.61% vs. 77.92%, P =0.008). In addition, the positive rates of DNA-based mNGS for bacteria, DNA viruses, and fungi were 68.66% (46/67), 72.73% (24/33), and 74.07% (20/27), while for CMTs were 98.51% (66/67), 81.82% (27/33), and 44.44% (12/27), respectively (**Figure 2C**). CMTs showed higher bacterial positive rates (CMTs vs. mNGS: 98.51% vs. 68.66%, P < 0.001; CMTs vs. culture: 98.51% vs. 86.57% P =0.013; **Figure 2C**). The fungal positive rates of DNA-based mNGS were higher than CMTs, but not statistically significant (mNGS vs. CMTs: 74.07% vs. 44.44%, P = 0.136, **Figure 2C**). The DNA viruses’ positive rates of DNA-based mNGS were comparable to CMTs (mNGS vs. CMTs: 72.73% vs. 81.82%, P = 0.332, **Figure 2C**)

In 25 RNA-based mNGS cases, the total positive rates of RNA-based mNGS, culture, and CMTs were to 80.00% (20/25), 52.00% (13/25), and 80.00% (20/25), respectively (**Figure 2D**). In addition, the positive rates of RNA-based mNGS for bacteria, DNA viruses, RNA viruses, and fungi were 80.00% (12/15), 100.00% (12/12), 50.00% (4/8), and 100.00% (5/5), while for CMTs were 93.33% (14/15), 83.33% (10/12), 100.00% (8/8), and 20.00% (1/5), respectively (**Figure 2D**). There were no significant difference between RNA-based mNGS and CMTs (all P > 0.05; **Figure 2D**).

### 3.4 Diversity of lung microbiome in pediatric pneumonia patients

Total 116 BALF samples from 104 pediatric pneumonia patients were sequenced by mNGS for the diversity and structure analysis of lung microbiomes.

**Figure 5:**
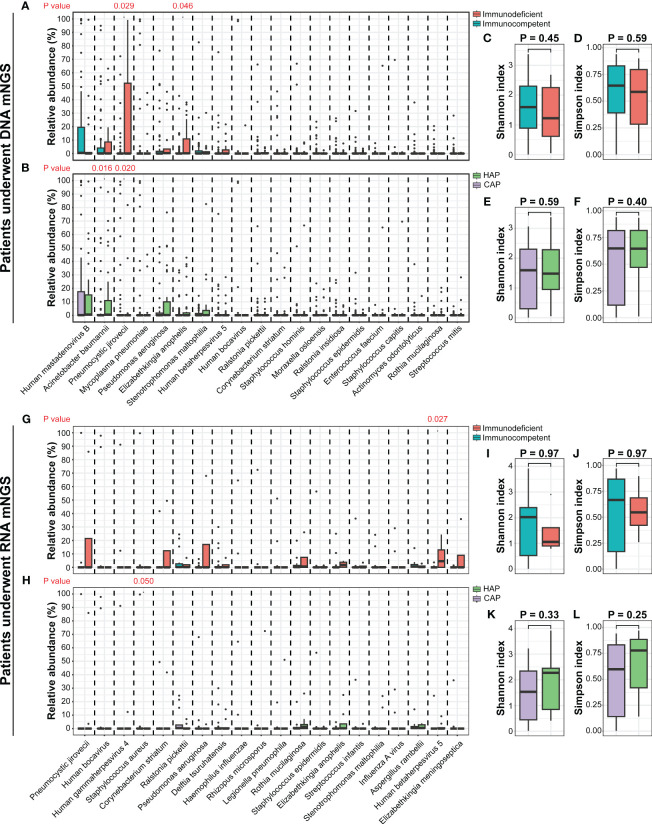
The comparisons of lung microbiomes among different pediatric pneumonia patient subtypes. **(A)** The relative abundances of the most abundant twenty species between patients with and without immunodeficiency who underwent DNA mNGS testing. **(B)** The relative abundances of the most abundant twenty species between patients carrying CAP and HAP who underwent DNA mNGS testing. **(C-F)** The comparisons of the Shannon and Simpson indexes among different pediatric pneumonia patient subtypes under DNA mNGS testing. **(G)** The relative abundances of the most abundant twenty species between patients with and without immunodeficiency who underwent RNA mNGS testing. **(H)** The relative abundances of the most abundant twenty species between patients carrying CAP and HAP who underwent RNA mNGS testing. **(I-L)** The comparisons of the Shannon and Simpson indexes among different pediatric pneumonia patient subtypes under RNA mNGS testing.

In 91 DNA-based mNGS samples, α-diversity was measured by the Shannon and Simpson index, representing the richness and evenness of the microbiome. There were no significant differences in the α-diversity between the CAP and HAP groups, and between immunodeficient and immunocompetent patients (**Figures 5C-F**). The relative abundance of prominent taxa across the patients with CAP and HAP and those with and without immunodeficiency were compared (**Figures 5A, B**, **Figures S1**, **2**). At the phylum level, there were no significant differences in the relative abundance of the most abundant ten phyla between the CAP and HAP groups (**Figure S1**), while *Firmicutes* (P=0.039) and *Proteobacteria* (P=0.045) was enriched in immunocompetent patients (P=0.036, **Figure S2**). At the genus level, the relative abundance of *Acinetobacter* was significantly higher in the HAP group (P=0.023), and *Pneumocystis* was significantly higher in the CAP group (P=0.014; **Figure S1**). *Pneumocystis* (P=0.036) were also significantly enriched in the immunodeficient patients (**Figure S2**). At the species level, the relative abundance of *Acinetobacter baumannii* (P=0.016) was significantly higher in the HAP group, while *Pneumocystis jirovecii* (P=0.020) were enriched in the CAP group (**Figure 5B**). *Pneumocystis jirovecii* (P=0.029) and *Elizabethkingia anopheles* (P=0.046) were significantly enriched in immunodeficient patients (**Figure 5A**).

In 25 RNA-based mNGS samples, α-diversity of Shannon and Simpson index were no significant difference between the CAP and HAP groups, and between the immunodeficient and immunocompetent patients (**Figures 5I-L**). The relative abundance of prominent taxa at species, genus, and phylum level across the patients with CAP and HAP and those with and without immunodeficiency were compared as well. (**Figures 5G, H**, **Figures S1**, **2**). At the species level, the relative abundance of *Staphylococcus aureus* (P=0.049) was significantly higher in the CAP group, while *Human betaherpesvirus 5* (P=0.027) were enriched in immunodeficient patients (**Figure 5H**).

### 3.5 Associations of microbial and clinical characteristics in pediatric pneumonia patients

**Figure 6:**
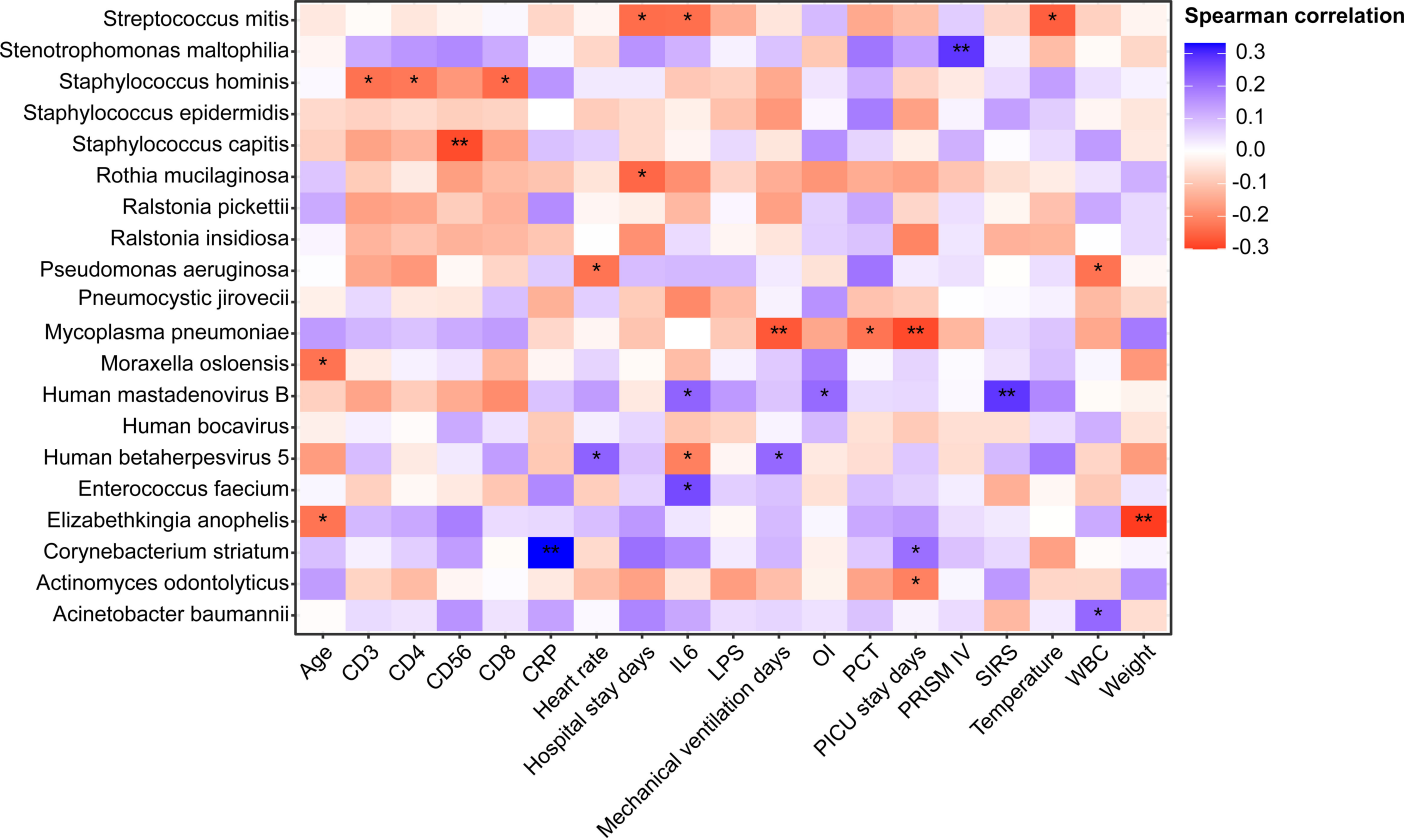
Heatmap of Spearman correlations between demographic data, clinical measures and lung microbiome species of patients underwent DNA mNGS testing. ^*^P < 0.05, ^**^P < 0.01 . OI, Oxygen index; SIRS, Systemic inflammatory response syndrome; CRP, C-reactive protein; WBC, White blood cell count; PCT, procalcitonin; IL-6, Interleukin-6; LPS, Lipopolysaccharide; PRISM, pediatric risk of mortality score.

Due to the small sample size of RNA-based mNGS, Spearman correlation analysis between clinical factors and the most abundant 20 species was performed in patients who underwent DNA-based mNGS testing, unveiling the relationships between lung microbiome, demographic data and clinical measures (**Figure 6**).

In 91 DNA-based mNGS samples, *Acinetobacter baumannii*, which was found to be more prevalent in patients with HAP, had a strong positive correlation with WBC, while *Pseudomonas aeruginosa* was negatively correlated to WBC. *Pneumocystis jirovecii*, which enriched in immunodeficient and CAP patients, was not identified associated with any clinical characteristics. *Corynebacterium striatum* had a strong positive correlation with PICU stay days. We also found that patients with high relative abundance of *Human betaherpesvirus 5* had longer mechanical ventilation days and higher heart rates. Notably, *Mycoplasma pneumoniae* had a negative correlation with PCT, PICU stay and mechanical ventilation days.

## Discussion

The value of BALF as being an excellent specimen for the etiologic diagnosis of pneumonia has been confirmed before (Chen et al., 2020). In this study, BALF specimens from 104 children were collected, and the etiology of pneumonia in the PICU was comprehensively described by combining mNGS and CMTs methods. Lung microecology was also analyzed using mNGS data, revealing the etiology of pneumonia from another perspective.

Combining mNGS and CMTs, the positive rate of clinical comprehensive etiology of PICU pneumonia was 94.06%, which was in line with previous reports with proportions of more than 90% (Deng et al., 2022; Guo et al., 2022; Tsitsiklis et al., 2022) and higher than the diagnosis rate reported without mNGS involvement (Zhu et al., 2018). Meanwhile 40.59% of patients changed their anti-infective treatment after implementing the mNGS results, close to the rate of 35.7% previously reported in children with pneumonia (Yang et al., 2022). mNGS combining with CMTs improved the diagnosis rate of pneumonia pathogenesis and provided a basis for the adjustments of anti-infective strategy, which will be an important direction for the future development of pneumonia diagnosis and treatment (Peng et al., 2021; Guo et al., 2022; Yang et al., 2022).

In 77 cases with DNA-based mNGS, the overall positive detection rate of mNGS was 81.82%, with a sensitivity of 85.14%, which was in the range of mNGS sensitivity reviewed by Chen et al (Chen et al., 2022). In the systematic review of mNGS to identify pathogens in BALF from patients with pulmonary infections, the pooled sensitivity was 78% (95% CI: 67-87%). Subgroup analyses of mNGS sensitivity demonstrated that severely or immunocompromised pulmonary-infected patients had a positive detection rate of 92% (95% CI: 78-100%) (Chen et al., 2022). Notably, in our study, no significant difference in the overall positive rates was identified, except between CMTs and culture. Patients in the PICU often have a history of hospitalization in other departments before admission, and CMTs typically include culture, PCR, and serology results obtained during the hospitalization for pneumonia, providing a more comprehensive overview of the pathogenic findings. CMTs demonstrated a significant advantage not only in the overall positive rate but also in the positive rates for bacteria, fungi, and viruses. Compared to culture, CMTs had advanced detection performance on atypical pathogens and RNA viruses, relying primarily on PCR and serological results, as atypical pathogens, fungi, and viruses that are difficult or impossible to culture. Although the overall positive rate of mNGS was higher than that of culture in this study, the gap was not as significant as in previous reports (Deng et al., 2022; Yang et al., 2022), and the overall positive rate of culture was also higher in our study. Additionally, the overall positive rate and sensitivity of mNGS was lower than CMTs in this study, which was contrary to other reports (Deng et al., 2022; Guo et al., 2022; Tsitsiklis et al., 2022; Yang et al., 2022). The diversity of specimen types used for culture and CMTs, as well as the higher sampling frequency in the PICU compared to the general ward, resulted in higher overall positive rates, particularly for bacteria. On the contrary, mNGS was not routinely performed due to its limited availability and high cost and was usually reserved for critical and diagnostically challenging cases during patients’ PICU stay. While the overall positive rate of mNGS may not have been outstanding, mNGS was able to detect difficult-to-detect pathogens that may have been missed by culture and CMTs methods, especially for fungi such as *Pneumocystis jirovecii*, rare bacteria like *Bordetella pertussis* and *Bordetella parapertussis*, and some viruses such as *Human bocavirus 1*. The merits of mNGS in such pathogens have been demonstrated in previous reports in both children and adults (Jiang et al., 2021; Guo et al., 2022; Liu et al., 2022; Wang et al., 2022; Zhao et al., 2022). The positive rates for fungi detection by mNGS were higher than those of culture and CMTs in this study although not significant (both P=0.136). This finding is consistent with some previous studies (Yang et al., 2021; Jin et al., 2022; Lin et al., 2022), which have suggested that mNGS is more sensitive than traditional culture-based methods for detecting fungal infections.

In this study, the factor accounting for the significant difference in fungal positivity between CMTs and mNGS in this study may be that *Pneumocystis jirovecii* was only detected by mNGS and *Candida* was only detected by CMTs. In addition, we noted that *Candida* accounted for 9.9% of the infections. We do not routinely consider the *Candida* as the pathogen when detected in respiratory specimens, such as a single sputum culture. But in the following two conditions, we consider the *Candida* a pathogen in retrospective studies: (i) *Candida* is detected in multiple sample type and/or by multiple diagnostic methods, and the corresponding treatment is effective; (ii) patients with at least 1 *Candida*-positive test result and are at high risk for *Candida* infection, and the corresponding treatment is effective. In our study, 9.9% (10/101) patients with pneumonia were *Candida*-positive, which is in line with some previous studies in pediatric pneumonia patients (18.75%, 21/112; 9.16%, 23/253) (Yang et al., 2022; Li et al., 2023). However, some other studies showed relative lower infection rates in pediatric pneumonia patients (2.1%, 2/96; 1%, 1/101) (Deng et al., 2022; Yu et al., 2023). This discrepancy may largely be due to differences in patient populations. Major risk factors for respiratory infection with *Candida* include exposure to antibiotics, critical illness, immunocompromised status, use of mechanical ventilation, and hospitalization or intensive care unit (Pendleton et al., 2017). Patients admitted to PICU may experience higher *Candida* infection rates than CAP patients due to frequent invasive procedures, long hospital stays, and intense usage of antibiotics. In all, the detection of *Candida* must always be interpreted within its clinical and microbiological context (Pendleton et al., 2017), such as multi-method or multi-specimen positive results of etiology (combining results of culture, BDG, and microscopic morphology tests; combining results from respiratory and peripheral blood samples), presence of *Candida* infection risk factors, etc.

In addition, the positive DNA and RNA viruses’ rates between mNGS and CMTs were not significantly different. This may be due to the fact that most of the viruses detected in this cohort were common viruses included in the CMTs approach, and therefore do not highlight the advantages of mNGS in detecting rare viruses.

Among most detected pathogens in this study, *Mycoplasma pneumoniae* and *Klebsiella pneumoniae* were frequently found in pediatric pneumonia (Marchello et al., 2016; Ning et al., 2017), while the co-infection of *CMV* and *Pneumocystis jirovecii* was common in immunocompromised cases (Liu et al., 2022). The higher percentage of *Acinetobacter baumannii, Pseudomonas aeruginosam*, and *Stenotrophomonas maltophilia* in this study may be related to the high ratio of HAP/VAP in PICU (Jean et al., 2020; Mourani et al., 2021). The high rate of mixed infections (79.21%) in PICU patients in this study is generally higher than those reported in pediatric pneumonia of 45.8% (Deng et al., 2022), which could be due to the characteristics of PICU, such as having more invasive operations, more intense anti-infective treatment, and longer hospital stays. The highest proportion of mixed infectious types was bacterial and viral mixed infections (36.63%), which is consistent with previous reports (Deng et al., 2022; Tran Quang et al., 2022). The etiological findings of different types of pneumonia were compared in this study. *Mycoplasma pneumoniae* and *Staphylococcus aureus* were common in CAP (Ning et al., 2017; Oumei et al., 2018), and *Mycoplasma pneumoniae* and *Legionella pneumophila* were the most common atypical pathogens (Marchello et al., 2016). In addition, the fungal detection rates were significantly higher in patients with immunodeficiency (55.56% vs. 25.30%, P =0.025) and comorbidities (40.30% vs. 11.76%, P =0.007). As reported before, respiratory fungal infection was a severe clinical problem, especially in patients with compromised immune functions (Li et al., 2019).

The human respiratory tract is home to niche-specific communities of microorganisms that may serve as a gatekeeper, preventing respiratory pathogens from colonizing the respiratory system. In addition, the respiratory microbiota is thought to play a role in the maturation and maintenance of respiratory physiology and immunity, helping to maintain homeostasis in the respiratory system (Man et al., 2017). Hence, in this study, we also analyzed the lung microbiome using BALF-mNGS data, including α diversity, species composition, and correlations between high-frequency species and clinical indicators. No significant differences were found in α diversity within the CAP and HAP groups, as well as within the groups with and without immunodeficiency. The lack of differences may be influenced by the timing of BALF sampling. Collection time varied depending on clinical diagnostic needs and was not consistent across all patients in the course of pneumonia. Additionally, this may indirectly reflect that the alterations in the lung microecology, due to the environment and medication administration, for patients with pneumonia admitted to the PICU may converge, leading to decreased overall diversity with pathogenic microorganisms occupying the main ecological niche (Dai et al., 2018). Airway microbiotas were reported associated with an increased (risk microbiota) or decreased (resilience microbiota) incidence and severity of acute respiratory infection in children (Hasegawa and Camargo, 2015). We found some pathogens like *Mycoplasma pneumoniae, Pneumocystis jirovecii, Elizabethkingia anopheles*, and *Acinetobacter baumannii* were strongly correlated with clinical characteristics. It was reasonable to assume that these species may be able to act as microbial markers indicating the severity and prognosis of pneumonia. It was to be expected that the relative abundance of *Pneumocystis jirovecii* was negatively correlated with immune cells counts, since immunodeficiency is identified as a risk factor for PJP. Interestingly, the PCT value and the relative abundance of *Mycoplasma pneumoniae* were found negatively correlated, in line with previous literature (Zhou et al., 2022), but the results and mechanisms need to be further validated. One study showed that PCT < 0.25 μg/L was statistically associated with *Mycoplasma pneumoniae* infection (Meyer Sauteur et al., 2020).

This study has several limitations. Firstly, it is a single-center retrospective study with a relatively early inclusion period, which may be limited by the maturity of mNGS technology and the timing of clinical use. The patient’s sleep state and the complexity of preparation before sampling may lead to a delay in BALF collection compared to conventional CMTs specimens. Secondly, PICU patients are mostly hospitalized patients who have deteriorated and transferred in, often already receiving anti-infective treatment, which may induce changes in the pathogenic infection. Finally, the comparison was between the mNGS results of BALF and the overall CMTs results during the patient’s hospitalization for pneumonia, so there were certain temporal and spatial differences, which may be the main reason why mNGS overall performance is not as good as CMTs. The mNGS technology itself needs to be optimized. For example, the need to reduce the interference of the human host genome, which can improve the sensitivity of microbial detection (He et al., 2022; Zhaoyang et al., 2022). Moreover, the subjectivity in the personalized interpretation of microorganisms detected in respiratory specimens may influence the result because the final diagnosis of the pathogenic microorganisms relies on the interpreter’s knowledge and experience in mNGS technology, medical microbiology, and infectious disease. It was proposed that a “clinical microbial sequencing board” should be established to discuss the mNGS results in the clinical context with medical providers (Stratton et al., 2021). Standardized processes and quality control of mNGS are also key points, which can directly affect the accuracy of mNGS results and the feasibility of comparing the results from different assays (Liu et al., 2021).

In summary, this study provided a detailed characterization of the etiology of pneumonia in PICU using mNGS technology, which revealed information on pathogenicity and lung microbiome characteristics. The results demonstrated that mNGS had advantages in detecting fungi, some DNA viruses, and specific atypical pathogens. Given the high acuity and critical nature of PICU patients, it is important to consider earlier timing of mNGS sampling and testing, while regular CMTs monitoring remains crucial. The combination of mNGS and CMTs can achieve high efficacy in pathogen testing, leading to faster diagnosis, improved medication regimens, and higher cure rates.

## Data availability statement

The datasets presented in this study can be found in online repositories. The names of the repository/repositories and accession number(s) can be found below: https://db.cngb.org/cnsa/ - CNP0004045.

## Ethics statement

The studies involving humans were approved by Ethics Committee of the Children’s Hospital of Fudan University. The studies were conducted in accordance with the local legislation and institutional requirements. Written informed consent for participation in this study was provided by the participants’ legal guardians/next of kin.

## Author contributions

HS and GY contributed to the conceptualization. HS and TL were responsible for data curation. MS and YZ performed the formal analysis. WC, HC, YW, JL, JT, and LH validated the results. HS and MS wrote the original draft. HS, MS and HC revised the manuscript. GL was responsible for admin and resources. GY contributed to the funding acquisition and study supervision. All authors have read and approved the final manuscript.
