# Supplementary material for: Utilizing metagenomic next-generation sequencing for diagnosis and lung microbiome probing of pediatric pneumonia through bronchoalveolar lavage fluid in pediatric intensive care unit: results from a large real-world cohort
Source: Front Cell Infect Microbiol. 2023 Aug 15;13:1200806. doi: 10.3389/fcimb.2023.1200806 (PMC10466250; doi:10.3389/fcimb.2023.1200806)
Supplement: Supplementary file 1 [file DataSheet_1.docx]

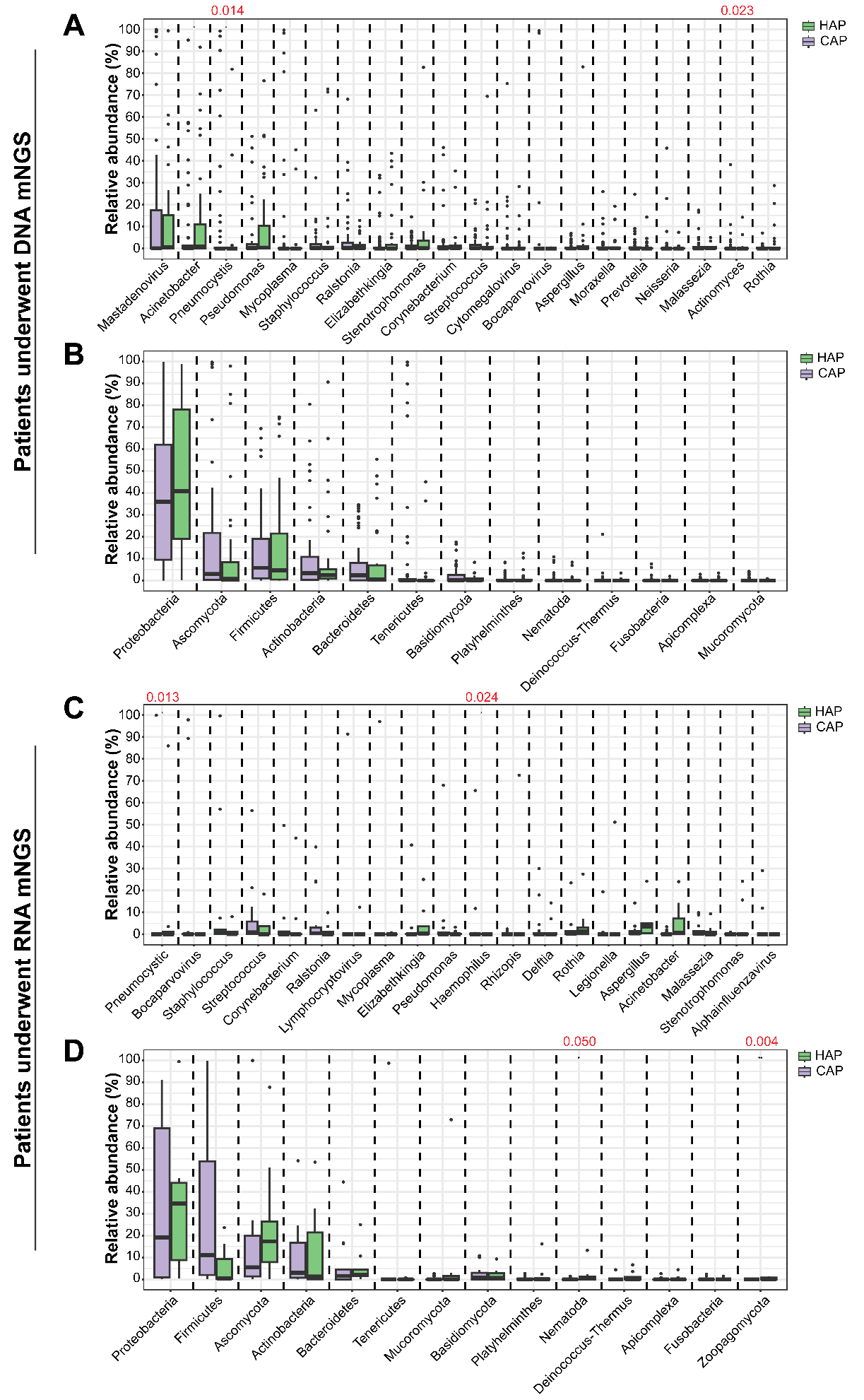


**Figure S1:** **The comparisons of lung microbiomes between patients carrying community-acquired pneumonia (CAP) and hospital-acquired pneumonia (HAP).** The differences in the relative abundances of the most abundant twenty species at (A) genus level, and (B) phylum level, between patients carrying CAP and HAP who underwent DNA mNGS testing. The differences in the relative abundances of the most abundant twenty species at (C) genus level, and (D) phylum level, between patients carrying CAP and HAP who underwent RNA mNGS testing.


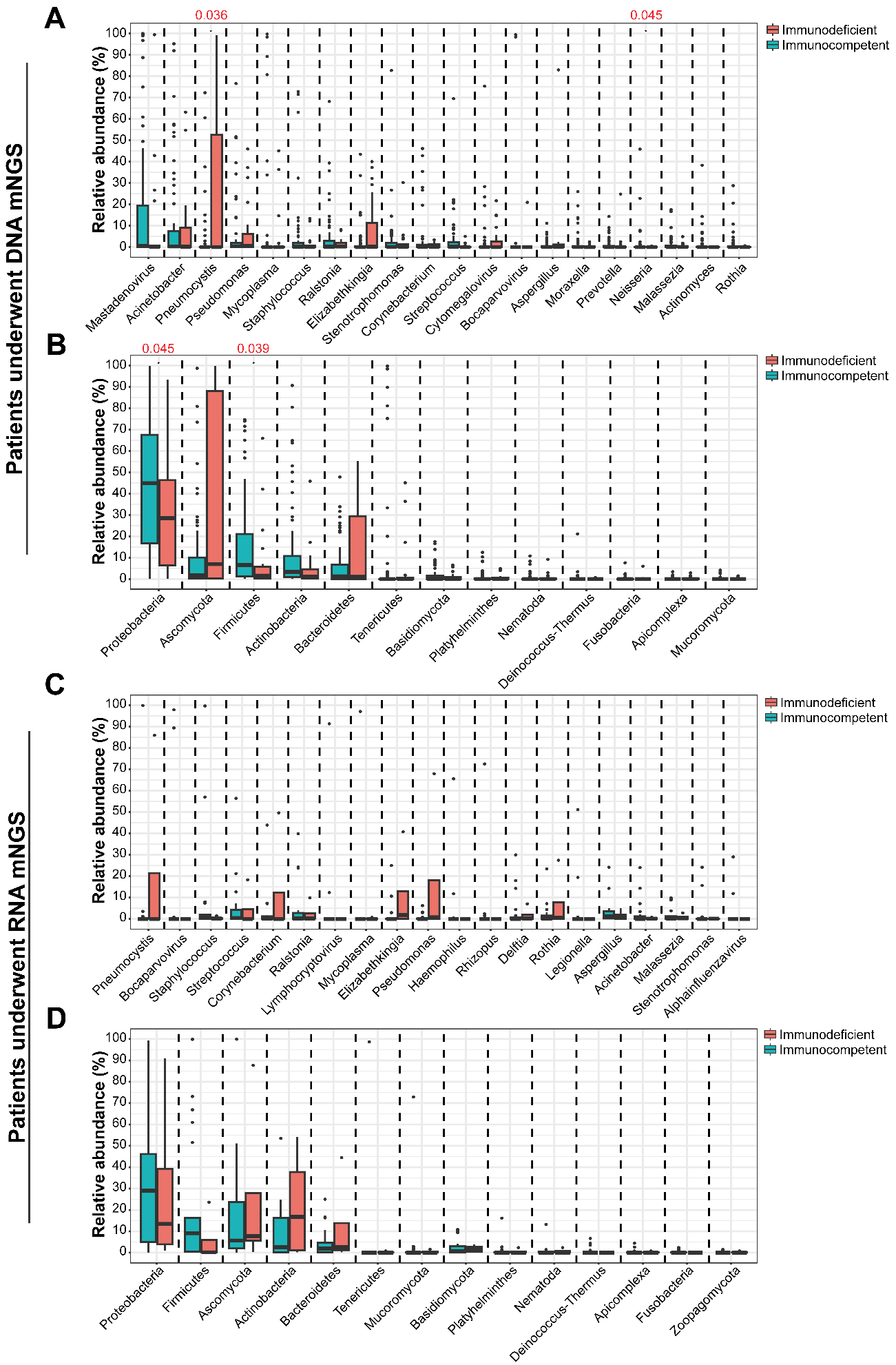


**Figure S2:** **The comparisons of lung microbiomes between immunodeficient and immunocompetent patients.** The differences in the relative abundances of the most abundant twenty species at (A) genus level, and (B) phylum level, between immunodeficient and immunocompetent patients who underwent DNA mNGS testings. The differences in the relative abundances of the most abundant twenty species at (C) genus level, and (D) phylum level, between immunodeficient and immunocompetent patients who underwent RNA mNGS testings.
